# Supplementary material for: A three-dimensional phase-field model for multiscale modeling of thrombus biomechanics in blood vessels
Source: PLoS Comput Biol. 2020 Apr 28;16(4):e1007709. doi: 10.1371/journal.pcbi.1007709 (PMC7224566; doi:10.1371/journal.pcbi.1007709)
Supplement: S1 Text — (PDF) [file pcbi.1007709.s001.pdf]

S1 Text. Governing equations in 2D

We summarize the governing equations for the 2D phase-field model as follows

$$
\begin{aligned}
&\rho(\frac{\partial \mathbf{u}}{\partial t} + \mathbf{u} \cdot \nabla \mathbf{u}) + \nabla p - \nabla \cdot (\eta(\phi) \nabla \mathbf{u}) + \nabla \cdot (\lambda_e \phi \nabla \psi^T \nabla \psi) \\
&+ \lambda \nabla \cdot (\nabla \phi \otimes \nabla \phi) + \eta(\phi) \frac{(1-\phi)\mathbf{u}}{\kappa} = 0, \\
&\nabla \cdot \mathbf{u} = 0, \\
&\frac{\partial \psi}{\partial t} + \mathbf{u} \cdot \nabla \psi = 0, \\
&\frac{\partial \phi}{\partial t} + \mathbf{u} \cdot \nabla \phi - \tau \Delta(-\lambda \Delta \phi + \lambda \gamma g_1(\phi) + \frac{\lambda_e}{2} |\nabla \psi|^2) = 0,
\end{aligned} \tag{1}
$$

where all the variables have the same notation as in the 3D model, whereas ψ is the auxiliary vector related to the deformation gradient tensor by $\mathbf{F} = \nabla \times \psi$.
