# Supplementary material for: A three-dimensional phase-field model for multiscale modeling of thrombus biomechanics in blood vessels
Source: PLoS Comput Biol. 2020 Apr 28;16(4):e1007709. doi: 10.1371/journal.pcbi.1007709 (PMC7224566; doi:10.1371/journal.pcbi.1007709)
Supplement: S2 Text — (PDF) [file pcbi.1007709.s002.pdf]

S2 Text. Thrombus permeability and deformation tests in a 2D channel

In this subsection, we conduct permeability and deformation tests in a 2D channel for the sake of completeness and to compare the simulation results with those in [1].

Thrombus permeability test in a 2D channel

First, we simulate the blood flow past a thrombus in a 2D channel and assume that the thrombus consists of a shell and a core, as shown by the green and blue regions in Fig. S1, with different permeability constants κ_s and κ_c, respectively. We consider two cases of different k_c as well as two cases of different surface tension σ to examine the sensitivity of the parameters employed in our model. The phase-field variable is fixed here, and the computational domain is $\Omega = \{(x, y)|\, 0 \leq x \leq 6, 0 \leq y \leq 2\}$. The thrombus is modeled by a half-circle located at the center of the channel. The inflow velocity at $x = 0$ has a parabolic profile with a maximum velocity of 1, whereas a zero-Neumann outflow condition is imposed at $x = 6$. No-slip boundary conditions are imposed at the walls.

We simulate three cases by varying the shell permeability $\kappa_s(\phi)$ and the height of the shell h_s, where we measure the total shear stress acting on the clot surface. Parameter values are included in Table A. We refer to the thrombus as the second phase and the blood as the first phase: $\rho_{1,2}$ and $\eta_{1,2}$ refer to the density and dynamic viscosity of the blood and the thrombus, respectively. Fig.S1 shows the streamwise velocity field u for case 3 with $h_s = 1.4$ and $h_c = 0.6$. We observe that upon increasing the shell permeability, the shear stress decreases monotonically. Furthermore, Fig.S2 shows that increasing the height of the shell h_s causes a sharper decrease of the shear force. These results agree with those in [1] and [2]. We calculate the shear stress on

the clot surface by the formula $F_\nu = |\int_S \mathbf{t} \cdot \eta(\nabla \mathbf{u} + \nabla \mathbf{u}^T) \cdot \mathbf{n}\, ds|$, where $\mathbf{t}$ and $\mathbf{n}$ are the tangential and normal directions of the interface, respectively.

Table A. Parameters used for the 2D channel permeability test in non-dimensional units.

	$\frac{\rho_2}{\rho_1}$	$\frac{\eta_2}{\eta_1}$	κ_c	σ	h_s	h_c
case 1	1	2	0.01	0.001	0.4	1.0
case 2	1	2	0.01	0.001	0.4	0.6
case 3	1	2	0.01	0.001	0.8	0.6

Thrombus deformation test in a 2D channel

Next, we use the same problem setup as before, but we vary the inflow velocity to investigate the effect of shear rates on thrombus deformation. The parameters are given in Table B. Fig.S3 shows different shapes of the thrombus under different shear rates at computational time $T = 2$. We observe in Fig.S3 that thrombus deformation becomes more pronounced as the shear rate increases.

Table B. Parameters used for the 2D channel deformation test in non-dimensional units.

**Parameters**	$\frac{\rho_2}{\rho_1}$	$\frac{\eta_2}{\eta_1}$	h_s	h_c	λ_e	σ
	2	1	1.2	0.6	0.5	0.01

References

1. Xu S, Xu Z, Kim OV, Litvinov RI, Weisel JW, Alber M. Model predictions of deformation, embolization and permeability of partially obstructive blood clots under variable shear flow. Journal of the Royal Society Interface. 2017;14(136):20170441.

2. Kim OV, Xu Z, Rosen ED, Alber MS. Fibrin networks regulate protein transport during thrombus development. PLoS Computational Biology. 2013;9(6):e1003095.
