# Supplementary material for: A three-dimensional phase-field model for multiscale modeling of thrombus biomechanics in blood vessels
Source: PLoS Comput Biol. 2020 Apr 28;16(4):e1007709. doi: 10.1371/journal.pcbi.1007709 (PMC7224566; doi:10.1371/journal.pcbi.1007709)
Supplement: S3 Text — (PDF) [file pcbi.1007709.s003.pdf]

S3 Text. Sensitivity tests of interface width & surface tension

There are four parameters in the governing equation, elastic shear modulus (λ_e), mixing energy density (λ), viscosity (η), and permeability (κ). We run two cases for the interface width (h), which dictates the magnitude of the mixing energy λ, and three cases for the surface tension (σ) to show that h and σ are not as important as the permeability (κ) and the viscoelasticity (λ_e). As for the interface width h and surface tension σ, [1] mentioned that the interface width h cannot exceed two cell widths for phase-field model calculations and the deformation of the thrombus largely depends on the permeability and the shear rate. Also, see Results section in this paper for the detailed discussion for the effects of the permeability and viscoelasticity on the deformation of the thrombus.

Table A and B include parameter values used. Figs.S4 and S5 show that, with different interface width and surface tension between fluid and thrombus phase, we have almost identical velocity and phase-field profiles.

Table A. Sensitivity of the interface width (h) in the 2D channel blood clot deformation test in non-dimensional units.

Parameters	$\frac{\rho_2}{\rho_1}$	$\frac{\eta_2}{\eta_1}$	h_s	h_c	λ_e	σ	h
	1	1	1.4	0.6	0	0.001	0.04
	1	1	1.4	0.6	0	0.001	0.045

Table B. Sensitivity of the surface tension (σ) in the 2D channel blood clot deformation test in non-dimensional units.

Parameters	$\frac{\rho_2}{\rho_1}$	$\frac{\eta_2}{\eta_1}$	h_s	h_c	λ_e	σ
	1	1	1.4	0.6	0	0.001
	1	1	1.4	0.6	0	0.0017
	1	1	1.4	0.6	0	0.002

References

1. Jacqmin D. Calculation of two-phase Navier–Stokes flows using phase-field modeling. Journal of Computational Physics. 1999;155(1):96–127.
