# Supplementary material for: A three-dimensional phase-field model for multiscale modeling of thrombus biomechanics in blood vessels
Source: PLoS Comput Biol. 2020 Apr 28;16(4):e1007709. doi: 10.1371/journal.pcbi.1007709 (PMC7224566; doi:10.1371/journal.pcbi.1007709)
Supplement: S4 Text — (PDF) [file pcbi.1007709.s004.pdf]

S4 Text. Simulation of thrombus deformation under steady inflow

We simulate the interaction between the blood and the thrombus under steady inflow, where all other parameters are the same as the pulsatile flow case except that the inflow is the parabolic shape with the velocity magnitude $0.059m/s$, see Fig.S6. As mentioned in [1], the unsteady flow produces a higher-pressure drop than the steady flow.

References

1. Banerjee, Moloy Kumar and Ganguly, Ranjan and Datta, Amitava. Effect of pulsatile flow waveform and Womersley number on the flow in stenosed arterial geometry. ISRN Biomathematics. 2012;.
