# Supplementary material for: A three-dimensional phase-field model for multiscale modeling of thrombus biomechanics in blood vessels
Source: PLoS Comput Biol. 2020 Apr 28;16(4):e1007709. doi: 10.1371/journal.pcbi.1007709 (PMC7224566; doi:10.1371/journal.pcbi.1007709)
Supplement: S5 Text — (PDF) [file pcbi.1007709.s005.pdf]

S5 Text. Modeling platelet transport and aggregation using FCM

The translational velocity of each platelet (passive or activated) is estimated by the local average of the fluid velocity weighted by a Gaussian kernel function. The governing equations for the incompressible flow are the mass balance (continuity) and linear momentum balance (Navier-Stokes) equations

$$\nabla \cdot \mathbf{u} = 0, \tag{1}$$

$$\rho(\frac{\partial \mathbf{u}}{\partial t} + \mathbf{u} \cdot \nabla \mathbf{u}) = -\nabla p + \mu \nabla^2 \mathbf{u} + \mathbf{f}(\mathbf{x}, t) \tag{2}$$

where $\mathbf{u}$, p, and μ are the flow velocity, pressure and blood viscosity, respectively, with body force $\mathbf{f}$ used to capture the effects of the particles (platelets) on the flow. Specifically,

$$\mathbf{f}(\mathbf{x}, t) = \sum_{n=1}^{N} \mathbf{F}^n \, \Delta(\mathbf{x} - \mathbf{Y}^n(t)) \tag{3}$$

$$\Delta(\mathbf{X}) = (2\pi\sigma^2)^{-3/2} \exp(-\mathbf{X} \cdot \mathbf{X}/2\sigma^2) \tag{4}$$

Here, $\mathbf{F}^n$ is the force due to particle n in the local region of interest. Moreover, the contribution of each platelet, whose center of mass is located at $\mathbf{Y}^n$, to the flow at position $\mathbf{x}$ is smoothed by a Gaussian distribution kernel $\Delta(\mathbf{X} \equiv (\mathbf{x} - \mathbf{Y^n}))$ with σ the standard deviation of the kernel, which is related to particle radius $a = (r_p$ or $r_{eff})$ through $\sigma = a/\sqrt{\pi}$. The governing equations are written in weak form and the domain is discretized using spectral elements that allow high order Jacobi polynomials. Time integration is performed using a semi-implicit splitting scheme [1]. The velocity of each platelet $\mathbf{V}^n$ is calculated by interpolating the local flow velocity at the location of a platelet using the same Gaussian kernel of kernel although different standard

deviations may be used for force and velocity interpolations. Specifically,

$$\mathbf{V}^n = \frac{d\mathbf{Y}^n}{dt} = \int \mathbf{u}\,\Delta(\mathbf{x} - \mathbf{Y}^n(t))d\mathbf{x} \tag{5}$$

where position vectors for all the platelets are updated at each time step using a second-order Euler forward scheme.

Further, the net force $\mathbf{F}^n$ acting on each platelet is written

$$\mathbf{F}^n = -\frac{4}{3}\pi a^3(\rho_{plat} - \rho_{fluid})\frac{d\mathbf{V}^n}{dt} + \mathbf{F}_{inter} \tag{6}$$

where the first term is the inertial force resulting from the difference in mass density between the platelets and blood flow and the second term accounts for interaction forces between platelets and the wall, which represent effects of different ligand-receptor interactions. Specifically, $\mathbf{F}_{inter} = -\partial U/\partial r$, where r is the distance between platelet centroids and the potential $U = U_{Morse} + U_{exp}$.

Specifically, we use a phenomenological model based on Morse potential U_{Morse}, having units of (Nm), to model attractive interactions amongst platelets, written as

$$U_{Morse} = D_e[1 - e^{-\beta d(r/d-1)}]^2 \quad r > d \tag{7}$$

where D_e is the energy depth contributing to the strength of the interaction force, β controls the width of the energy well, and $d = 2r_p$ (or $2r_{eff}$ if activated) is the equilibrium distance between two platelets. To avoid instability due to strong Morse repulsive forces, we use an exponential repulsion potential in the form of

$$U_{exp} = \frac{A}{1 - e^{Br_c}}[e^{Br_c}r - \frac{e^{Br}}{B}] \quad r < d \tag{8}$$

where the parameter A defines the maximum repulsive strength as $r \to 0$, B is an adjustable parameter that defines the shape of the repulsive force, and r_c is the cutoff radius. By setting B to a large positive number (here $B = 25$ in nondimensional units) and letting $r_c = d$, we can obtain a hard-sphere repulsive potential. We set the parameter A such that the ratio of maximum repulsive to attractive forces is 5. Note that unlike the standard Morse repulsion, the exponential repulsion does not become

singular as $r \to 0$, which eliminates potential instabilities in particle systems.

The value of βd defines the range of interactions between platelets; it is selected to be 2.5 to prevent overlap of passive platelets though partial overlap is allowed for activated platelets ($F_{rep,max}/|F_{att,max}| = 5$). The Morse attractive force is negligible for $r/d > 3$, which is considered as the cutoff radius for the attractive forces to avoid long-range interactions. The undetermined parameter D_e, which controls the magnitude of platelet interaction forces, and r_{eff}, which controls the number of particles in the simulation are calibrated in [2]. To calibrate the platelet aggregation model, we consider an interaction distance of $2d$ between platelets within which resting platelets may become activated.

References

1. Karniadakis G, Sherwin S. Spectral/hp element methods for computational fluid dynamics. 3rd ed. Oxford University Press; 2013.

2. Yazdani A, Li H, Humphrey JD, Karniadakis GE. A general shear-dependent model for thrombus formation. PLoS Computational Biology. 2017;13(1):e1005291.
