# Supplementary figures and images for: A three-dimensional phase-field model for multiscale modeling of thrombus biomechanics in blood vessels
Source: PLoS Comput Biol. 2020 Apr 28;16(4):e1007709. doi: 10.1371/journal.pcbi.1007709 (PMC7224566; doi:10.1371/journal.pcbi.1007709)

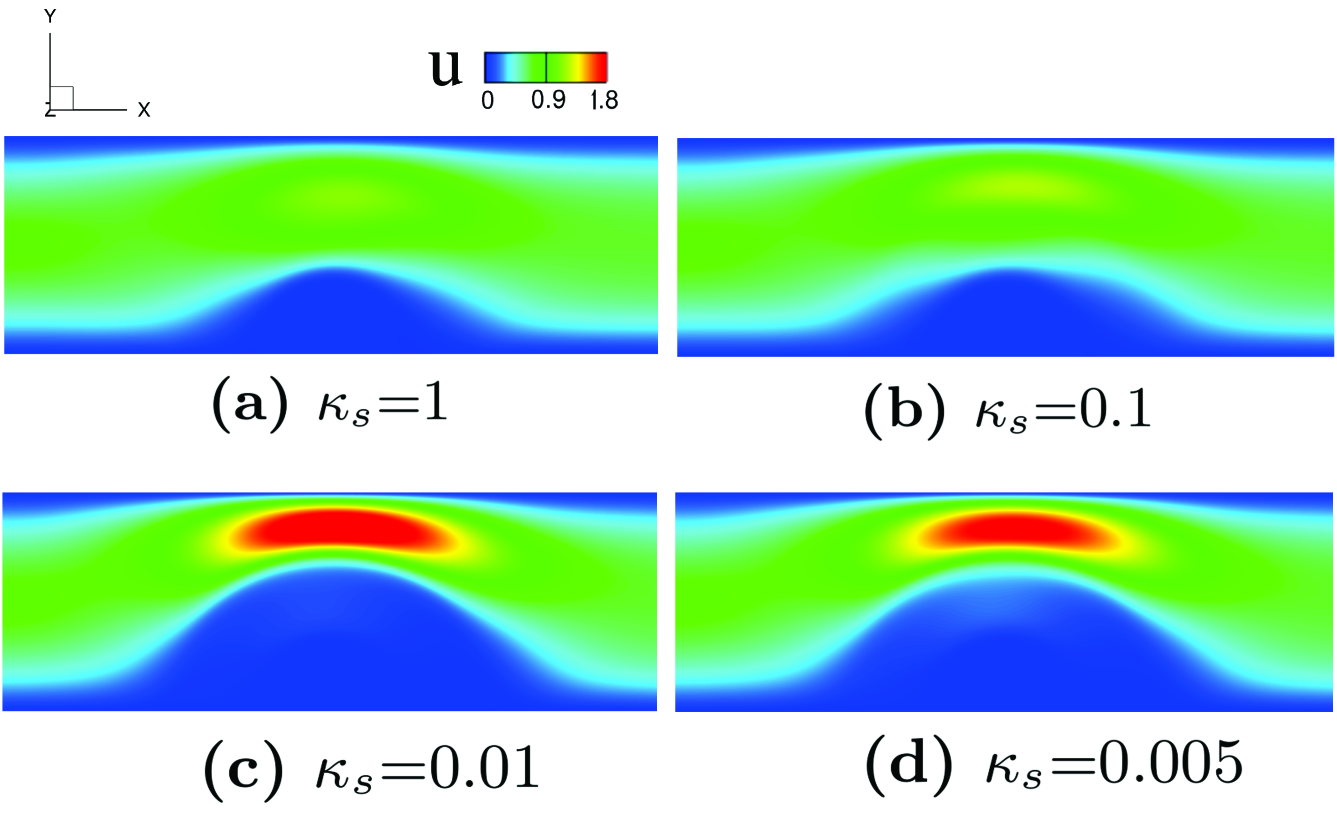

Supplement: S1 Fig — Stream-wise velocity field u for case 3 (see Table 1) with hs = 1.4 and hc = 0.6 at (a) κs = 1, (b) 0.1, (c) 0.01, and (d) 0.005 shown at time T = 0.7. (TIFF) [file pcbi.1007709.s006.tiff]

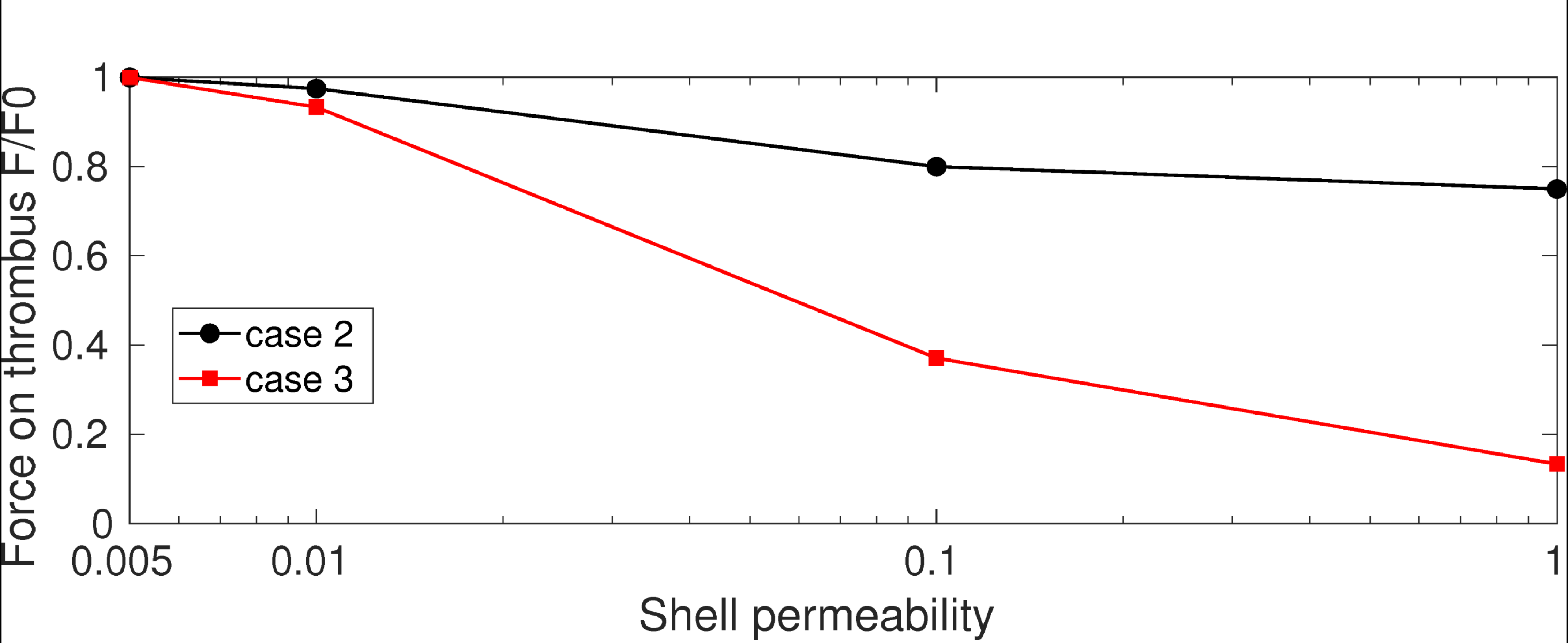

Supplement: S2 Fig — Shear stress (normalized by the stress F0 calculated with κs = 5e − 3) acting on the surface of the thrombus as a function of κs, with case 2 (black line) and case 3 (line) corresponding to different sizes of shell sub-domain of the thrombus. (TIFF) [file pcbi.1007709.s007.tiff]

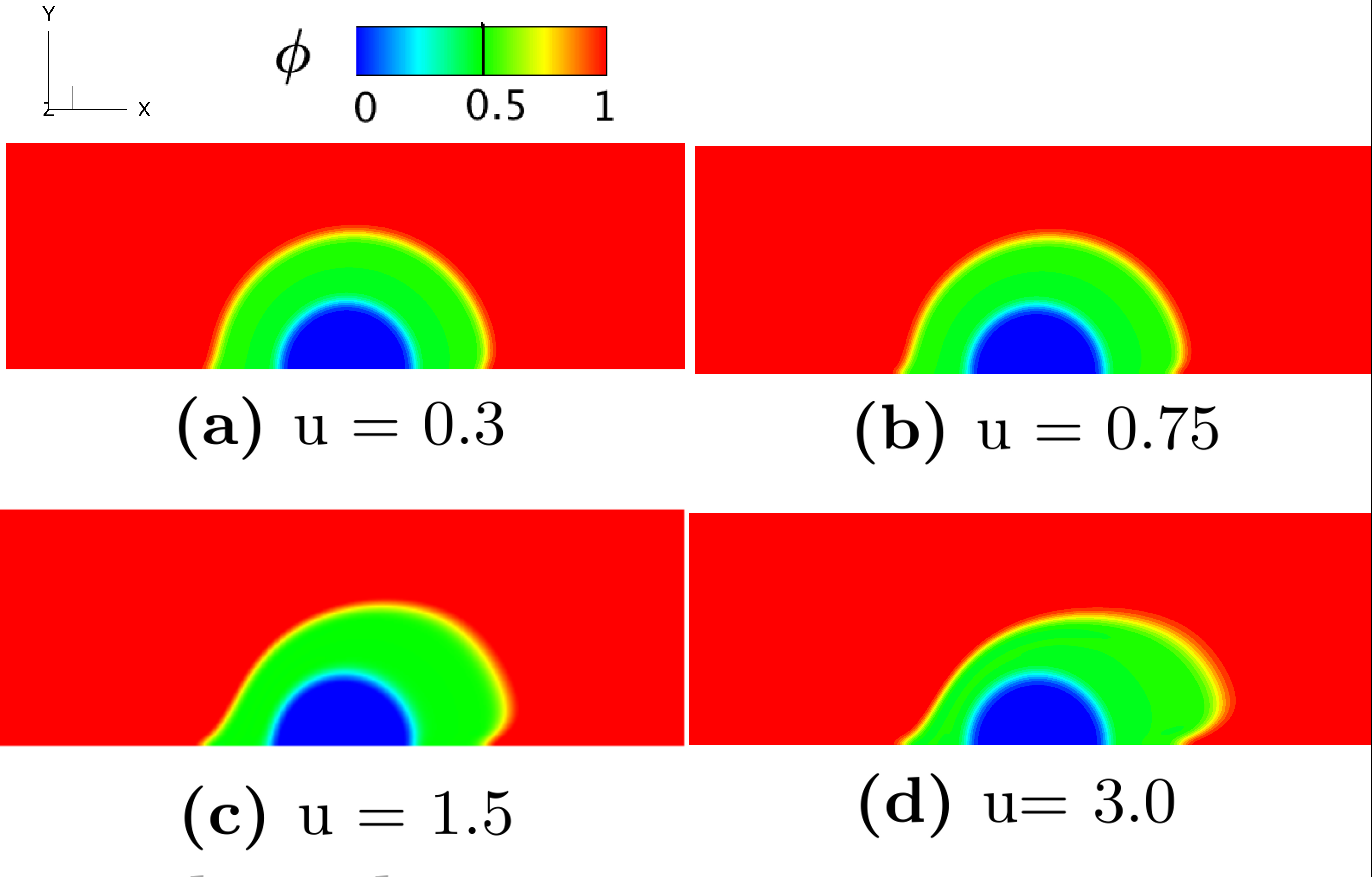

Supplement: S3 Fig — Impact of the flow velocity u on thrombus deformation with hs = 0.6 and hc = 0.3. Phase-field contour of the thrombus at different shear rates are plotted for (a) u = 0.2, (b) 0.5, (c) 1.0 and (d) 2.0 at time T = 0.48. u is the maximum velocity at the inlet. (TIFF) [file pcbi.1007709.s008.tiff]

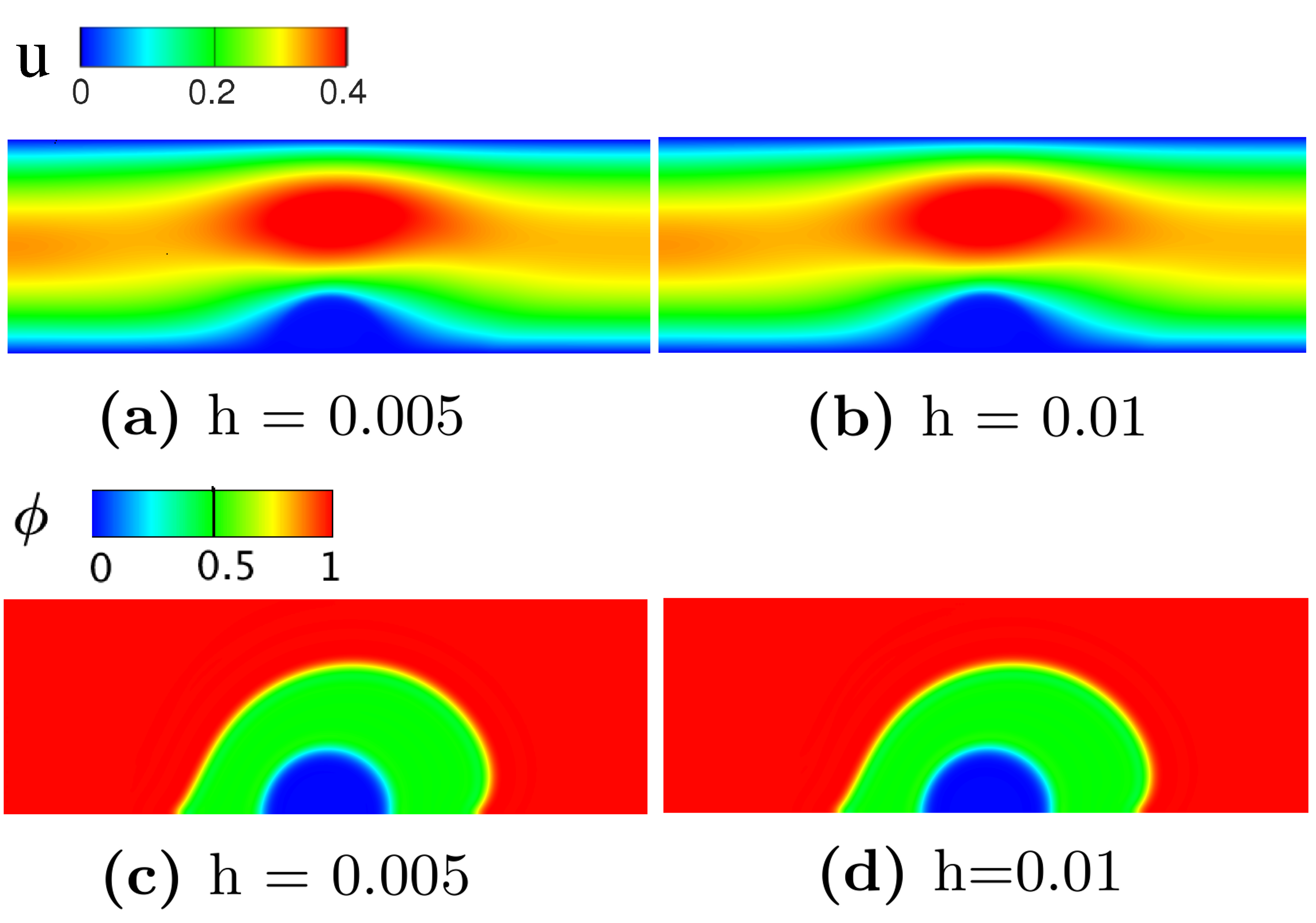

Supplement: S4 Fig — 1st row: axial velocity and 2nd row: phase-field profile at T = 1. h is the interface width. (TIFF) [file pcbi.1007709.s009.tiff]

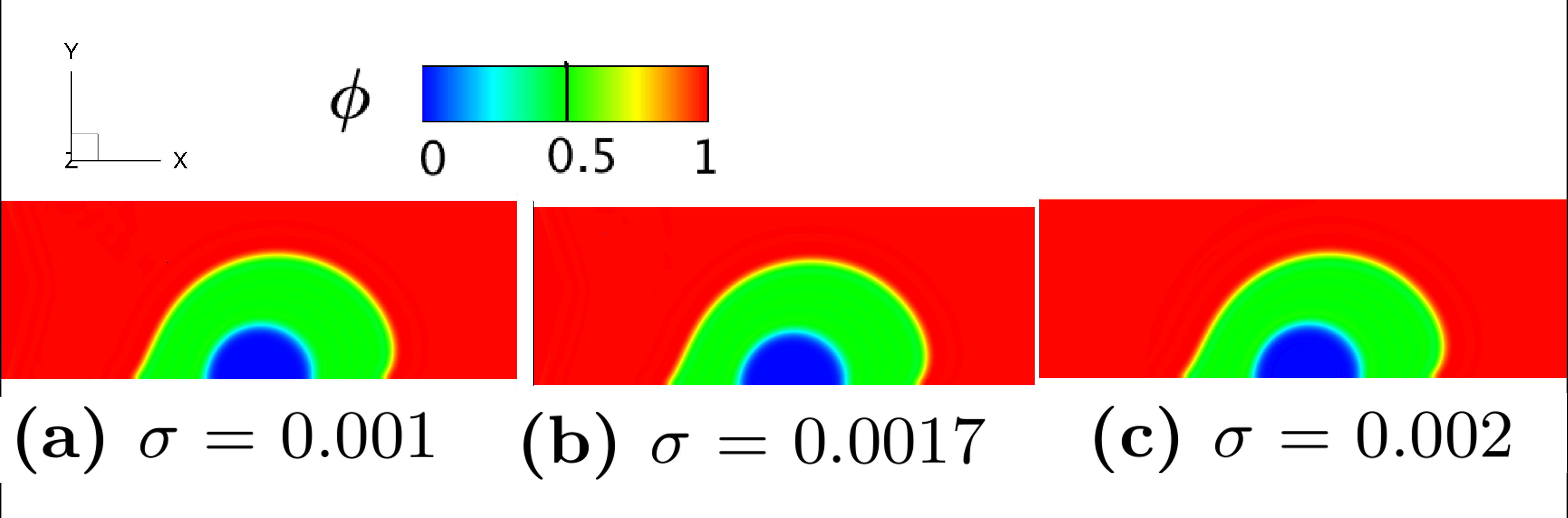

Supplement: S5 Fig — Phase-field profile at T = 1. σ is the surface tension. (TIFF) [file pcbi.1007709.s010.tiff]

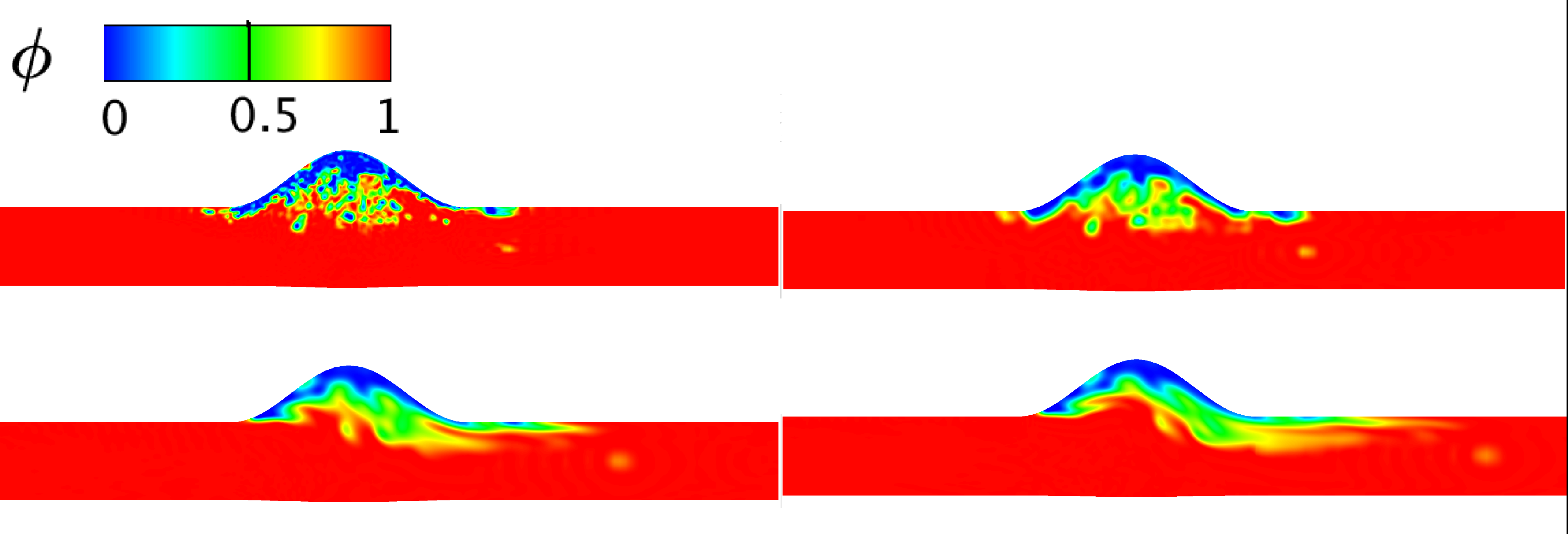

Supplement: S6 Fig — (TIFF) [file pcbi.1007709.s011.tiff]
