# Supplementary material for: A three-dimensional phase-field model for multiscale modeling of thrombus biomechanics in blood vessels
Source: PLoS Comput Biol. 2020 Apr 28;16(4):e1007709. doi: 10.1371/journal.pcbi.1007709 (PMC7224566; doi:10.1371/journal.pcbi.1007709)
Supplement: Attachment — Submitted filename: PCOMPBIOL-D-19-01313_Review.pdf [file pcbi.1007709.s012.pdf]

Review of "A Three-dimensional Phase-field Model for Multiscale Modeling of Thrombus Biomechanics in Blood Vessels"

In this work, Zheng et al. developed a three-dimensional phase-field model for studying fibrin-based thrombus interaction with blood flow. Both the permeability and the viscoelasticity (shear modulus) of the clot are incorporated into the model and validated against existing in vitro data. Moreover, the authors applied the phase-field model to study the thrombus deformation under flow, where the initial morphology and microstructure of the thrombus are constructed using a continuum/particle force coupling method (FCM) that was developed previously.

General comments:

Regarding the methods, the phase-field based continuum approach informed by particle-scale modeling information is a novel methodology that attempts to bridge the clot microstructure and clot macroscopic properties. The model also seems to be correctly implemented and validated.

1. There are some notation inconsistencies that should be cleaned up. (see below)
2. The writing and analysis of the results are weaker. The text of methodology and literature justification which should be in the Methods section.
3. The text often fails to state what the results actually show, but merely point to Figures.
4. The results are often not related to physiologic dimensions so fail to describe the relationship of reported graphs to physiologic mechanisms.
5. Modeling can be used to explore the sensitivity of findings to assumed parameters, pointing out controlling parameters versus relatively inconsequential variants. The authors do not explore these aspects, such as whether pulsatility has any material effects on a clinical thrombus that may form over days to months.
6. The authors should also reflect on whether future experiments could validate or disprove the numerical predictions
7. and whether the results have any clinical utility.

Specific comments:

8. **In the Abstract (and throughout the paper):**

   The authors claim they "seamlessly integrate" the FCM model with the phase-field model. However, I believe, the FCM model was only used to form the thrombus structure providing the initial condition for the following phase-field modeling, i.e., the two modeling process were performed separately without any coupling. Therefore, a "pre-conditioning" type of modeling strategy should be claimed instead of claiming "seamless integration" of the two models.

   Introduction. The authors mix up several concepts in the understanding of the biology of thrombosis formation. A. Thrombus can be formed by several distinct mechanisms (e.g. Cadroy, 1989): a. coagulation from Virchow's triad that forms red clots under low shear, where no platelets are involved except as bystanders; or b. platelet aggregation at high shear that forms white clots. The role of shear, fibrin, thrombin, and platelet activation is quite different for each. Venous thrombus and maturity are poorly defined. They should state explicitly how their model reflects one or the other, or where experimental evidence may indicate both exist.

9. In Section 2.1:

a) The authors mentioned "After activation, pseudo-platelets (…) grow to …". This is a legitimate course-graining approach; however, shouldn't the pseudo-platelets grow after more platelets adhere instead just after activation? For completeness, the authors should also briefly mention how the platelet-platelet interaction forces (through Morse potential) were validated and whether it is shear-dependent or not; instead of simply referring to the previous publications.

b) The equation defining the local volume fraction of platelet particles needs more clarification.

c) The time scale for platelet activation of <0.3s is at odds with experiments that typically measure this in >2 min. e.g. Moake et al. and many in the 80s and 90s.

10. In Section 2.2:

a) The authors define the phase field variable as ϕ, where $\phi = 1$ denote fluid (blood) and $\phi = 0$ solid (thrombus). This can be very easily mixed up with the notation for volume fraction throughout the paper. In fact, later in section 3.2 paragraph 1, the authors reuse ϕ to denote volume fraction. This needs to be addressed since this causes a lot of confusion while reading the paper.

b) What is the definition of h in equation $g_1(\phi)$ and later λ?

c) Below equation (2e), the notation for velocity ($\boldsymbol{u}$) is missed.

11. In section 3.1:

a) The authors show the 3-D simulation with assuming periodic boundary condition in the third direction (z) can reduce to a 2-D problem. What is the point of this case for the methodology of the paper is based on a 3-D implementation and even following results are all based on 3-D simulation. Besides, assuming z direction follows periodic boundary in the specific case naturally implies the 3-D setup for this problem can degrade to a 2-D case. Instead, the authors should explain how the results are different and what we learn by adding complexity.

12. In section 3.2:

a) In the case of validating the permeability calculation, the authors should stress the fact that only fibrin (no platelets) is considered in this validation case since the case is to compare with the fibrin gel. Therefore, it might be over-stated to call this section as the validation of the permeability of the thrombus.

b) In the case of validating the permeability calculation, what was the justification of the initial distribution of the phase field variable (figure 7) selected for each volume fraction of fibrin clot?

c) In the section of "Calibration of thrombus shear modulus", the definitions of A_r and ΔT_s are not provided.

d) In the last paragraph of this section, the first and third λ_e should be λ_s instead.

e) VF also needs to be denoted in the G' and G'' plots since VF is used in the text while only the mass concentration is denoted in the plots.

f) What is the justification of selecting $\lambda_e = 0.44$ as the value of the clot shear modulus?

13. In section 3.3:

a) The authors assume the platelets will not accumulate on the clot or the wall after the initial platelet aggregation modeled by FCM. What is the justification of this assumption?

b) In Figure 12 (b), since in phase-field results $\phi = 1$ (red) denotes fluid phase, why do we see red regions for the thrombus location in the bottom figure as oppose to the blue ($\phi = 0$) regions shown in the top figure?

c) In Figure 13, what is the definition of T? Can the authors also list the physical time unit instead of only the nondimensional time?

d) Can the authors discuss or comment on the effects of the pulsatility on the formation of the thrombus and the deformation of the formed thrombus?

14. Discussion section 4.

In general, the authors should provide some analysis of the Results and put them in the context of thrombosis. For example:

a) How does the phase-field model predict thrombosis that is different from previous methods? Can these results be verified in an experiment?
b) Recent reports indicate that high shear thrombi are much more permeable than coagulation fibrin clots (Kobayashi ISTH 18). Does the inclusion of permeability in your model cause a large difference in forces on the clot or propensity to evolve differently?
c) Does the inclusion of a deformable thrombus create embolization where none existed before?
d) Figures 12 and 13 suggest large heterogeneity in thrombus formation in the first 15 seconds, yet Figure 11 indicates that the aneurysm will merely fill in with fibrin after a minute. Since the calculation of Fig 12 and 13 comes at great cost, should we abandon this detail?
