# Supplementary material for: A three-dimensional phase-field model for multiscale modeling of thrombus biomechanics in blood vessels
Source: PLoS Comput Biol. 2020 Apr 28;16(4):e1007709. doi: 10.1371/journal.pcbi.1007709 (PMC7224566; doi:10.1371/journal.pcbi.1007709)
Supplement: Attachment — Submitted filename: Review_for_phase_field.pdf [file pcbi.1007709.s013.pdf]

Answers to the reviewers' questions for PCOMPBIOL-D-1901313

ANSWERS FOR REVIEWER #1

We would like to thank **Reviewer #1** for the insightful comments that helped us to improve our manuscript. Please see below our answers to your report and questions.

1. There are some notation inconsistencies that should be cleaned up.

Answer: The notations have been revised to be consistent throughout the manuscript.

2. The writing and analysis of the results are weaker. The text of methodology and literature justification which should be in the Methods section.

Answer: We have revised the text in the Results section to strengthen the connection between the biomechanical properties, deformation of the clots, and the hemodynamics. We have moved the text related to the methodology to Methods section on page 18 and 19 in the revised manuscript.

3. The text often fails to state what the results actually show, but merely point to Figures.

Answer: We have added the text in the Results section to further analyse the results.

4. The results are often not related to physiologic dimensions so fail to describe the relationship of reported graphs to physiologic mechanisms.

Answer: We have revised the model calibration and the application sections such that all the simulation results are reported in the form of physical units. We also connect our results with physiologic mechanisms in the Results section on page 13 and the third paragraph of the Discussion section on page 16 in the revised manuscript.

5. Modeling can be used to explore the sensitivity of findings to assumed parameters, pointing out controlling parameters versus relatively inconsequential variants. The authors do not explore these aspects, such as whether pulsatility has any material effects on a clinical thrombus that may form over days to months.

Answer: We have added a section in Supporting information to discuss the sensitivity of model param-

eters. Our simulations of the thrombus deformations with different model parameters, listed in Tables 1 and 2 in the main text, as well as Tables 3 and 4 in Supporting information, show that the shear modulus and permeability are the two key parameters for our simulation results compared to other parameters, such as surface tension, density, and viscosity of the thrombi. We also added a simulation case with a steady inlet velocity to further investigate the interactions between the blood flow and thrombi in an idealized aneurysm in Supporting information. Our simulation results show that a thrombus under steady flow does not release emboli, which is different from the case of pulsative flow shown in Fig 13 in the main text. This difference suggests that pulsatility of the blood flow could contribute to embolism resulting from the thrombosis in aneurysms.

6. The authors should also reflect on whether future experiments could validate or disprove the numerical predictions.

Answer: We would like to emphasize that the parameters implemented in this framework can be refined with new experimental data, such as permeability and viscoelastic properties of thrombus with varying compositions of the fibrin, platelets, and erythrocytes. We have added this in the third paragraph of the Discussion section.

7.whether the results have any clinical utility.

Answer: By using patient-specific vessel geometries as well as the blood flow and blood composition, this model can be used to predict the risk of clot formation in an aneurysm and embolism for a specific patient. We have emphasized this in the fourth paragraph of the Discussion section.

8. The authors claim they "seamlessly integrate" the FCM model with the phase-field model. However, I believe, the FCM model was only used to form the thrombus structure providing the initial condition for the following phase-field modeling, i.e., the two modeling process were performed separately without any coupling. Therefore, a "pre-conditioning" type of modeling strategy should be claimed instead of claiming "seamless integration" of the two models.

Answer: We agree with the reviewer that the FCM (particle) solver was used in advance to allow the thrombus formation and subsequently, and the thrombus size and composition were then used as inputs to the phase-field solver. We have changed the terminology in the revised manuscript to "combine" in the

abstract.

8.2 The authors mix up several concepts in the understanding of the biology of thrombosis formation. A. Thrombus can be formed by several distinct mechanisms (e.g. Cadroy, 1989): a. coagulation from Virchow's triad that forms red clots under low shear, where no platelets are involved except as bystanders; or b. platelet aggregation at high shear that forms white clots. The role of shear, fibrin, thrombin, and platelet activation is quite different for each. Venous thrombus and maturity are poorly defined. They should state explicitly how their model reflects one or the other, or where experimental evidence may indicate both exist.

Answer: We thank the reviewer for raising this important point. As the reviewer points out the fibrin and platelet-rich white thrombi are often associated with atherosclerosis and develop in short timescale whereas the fibrin and erythrocyte-rich red thrombi are associated with low pressure systems such as in veins and develop over longer time scales. The composition of the thrombus in an aneurysm is complex. For example, in abdominal aortic aneurysms (AAAs), the thrombus may contain layers with different compositions, representing aggregation of blood cells at different time scales [1] and similarly for the thrombi found in the aortic dissecting aneurysms [2].

In this work, we model the formation of thrombus in an idealized AAAs, in which the low shear rate region inside the aneurysm combined with a long shear history experienced by the platelets promotes the deposition of the platelets [3, 4]. Instead of predicting the composition of the thrombi, we aim to demonstrate that the proposed framework can simulate the formation of thrombus that is initiated via platelet activation, which may develop to thrombi with complex (often layered) composition. We emphasize this in the Discussion section of the revised manuscript.

9. In Section 2.1: a) The authors mentioned "After activation, pseudo-platelets (...) grow to ...". This is a legitimate coarse-graining approach; however, shouldn't the pseudo-platelets grow after more platelets adhere instead just after activation? For completeness, the authors should also briefly mention how the platelet-platelet interaction forces (through Morse potential) were validated and whether it is shear-dependent or not; instead of simply referring to the previous publications.

Answer: In the application of our framework as shown in Figs 11 and 12, the volume of the idealized aneurysm is $\sim$7.3 cm^3, which is supposed to be filled with 1.095-3.285$\times 10^9$ platelets physiologically based on

the platelet number density of 150,000-450,000 per microliter of blood. Due to the large number of platelet particles involved, we cannot model all the platelet particles at their physiological size. Therefore, we simulate a *final-value* problem in lieu of the original *initial-value* problem with significantly fewer particles initially placed in the system which may grow in size upon activation, thus representing the local concentration of blood-borne species. This approach allows us to use fewer platelets than the physiologic concentration and to grow the size and shape of the clot.

The interactions between the platelets as well as between the platelets and the deposition sites are described by a Morse potential (attractive interactions between platelets) and an exponential repulsion potential (exclusive effects of the platelet particles). The interaction force are shear-rate dependent and the force values were validated by using data from four independent experimental studies, including two *in vivo* [5, 6] and two *in vitro* [7, 8] experiments, which measured platelet aggregation at different shear rates. More details about the FCM model were added in Methods section of the revised manuscript and the last section of Supporting information.

b) The equation defining the local volume fraction of platelet particles needs more clarification.

Answer: We have revised the third paragraph of Methods section to clarify each term in the definition of local volume fraction of the thrombus.

c) The time scale for platelet activation of ≤ 0.3s is at odds with experiments that typically measure this in ≥ 2 min. e.g. Moake et al. and many in the 80s and 90s.

Answer: The platelet delay time in our model is a parameter which is used to represent the effect of coagulation cascade pathways that activate the platelets, see [9, 10]. Therefore, it is fundamentally different from the physical delay time for platelets to become activated.

10. In Section 2.2: a) The authors define the phase field variable as ϕ, where $\phi = 1$ denote fluid (blood) and $\phi = 0$ solid (thrombus). This can be very easily mixed up with the notation for volume fraction throughout the paper. In fact, later in section 3.2 paragraph 1, the authors reuse ϕ to denote volume fraction. This needs to be addressed since this causes a lot of confusion while reading the paper.

Answer: In this work, the phase-field valuable is defined based on the volume fraction of the mixture, instead of the mass fraction. Therefore, the phase-field variable here is equivalent to the volume fraction in

this study, similar to the definition in prior studies [11, 12].

b) What is the definition of h in equation $g'(\phi)$ and later λ?

Answer: Following prior work [13] and [14], h is defined as the interface width and λ is defined as the mixing energy density. These definitions are in the second paragraph of Methods section on page 20.

c) Below equation (2e), the notation for velocity (u) is missed.

Answer: The notation for velocity (u) is added below equation (5e) on page 21 in the revised manuscript.

11. In section 3.1: a) The authors show the 3-D simulation with assuming periodic boundary condition in the third direction (z) can reduce to a 2-D problem. What is the point of this case for the methodology of the paper is based on a 3-D implementation and even following results are all based on 3-D simulation. Besides, assuming z direction follows periodic boundary in the specific case naturally implies the 3-D setup for this problem can degrade to a 2-D case. Instead, the authors should explain how the results are different and what we learn by adding complexity.

Answer: We conducted 3-D simulations with periodic boundaries to verify that our 3-D model are consistent with 2-D simulation results in the literature [15]. This is not a trivial verification as the mathematical formulation in 2D and in 3D are completely different. We can not use a 2-D model to simulate blood vessels because of inflow/outflow boundary conditions and complex artery geometries.

12. In section 3.2: a) In the case of validating the permeability calculation, the authors should stress the fact that only fibrin (no platelets) is considered in this validation case since the case is to compare with the fibrin gel. Therefore, it might be over-stated to call this section as the validation of the permeability of the thrombus.

Answer: There are two clot properties that have to be calibrated in our model: permeability and viscoelasticity. We have not been able to find any experiments that measures the viscoelasticity of platelet-rich clot, other than reference [55] which only considered fibrin gel only. Thus, we calibrated both the permeability and viscoelasticity of the model against fibrin gel for consistency. As a result of this approximation, the permeability of the thrombus model could be overestimated. We have performed a sensitivity study on the permeability of thrombus, which is shown in Fig 1 in Supporting information. Clearly, this approach is also valid for platelet-rich clot when experimental data is available. We have mentioned this limitation in

the Discussion section, see the third paragraph of the Discussion section in the revised manuscript.

b) In the case of validating the permeability calculation, what was the justification of the initial distribution of the phase field variable (figure 7) selected for each volume fraction of fibrin clot?

Answer: As the initial distribution of the volume fraction in the experimental study was unknown, here we assume that the volume fractions of the clots are uniformly distributed. As mentioned in response to question #10, the phase-field variable is equivalent to the volume fraction and thus the distribution of the phase-field variable represents the volume fraction of the fibrin clot roughly.

c) In the section of "Calibration of thrombus shear modulus", the definitions of A_r, δ, T_s are not provided.

Answer: We have added the definitions of these three parameters on page 12 in the revised manuscript.

d) In the last paragraph of this section, the first and third λ_e should be λ_0 instead.

Answer: The notations for the relaxation time λ_s are fixed in the revised manuscript.

e) VF also needs to be denoted in the G' and G" plots since VF is used in the text while only the mass concentration is denoted in the plots.

Answer: VF can be calculated from the mass concentration $c_{fg,0}$, which is the variable on the x-axis in Fig.7(i) in the revised manuscript.

f) What is the justification of selecting $\lambda_e = 0.44$ as the value of the clot shear modulus?

Answer: When λ_e is selected to be 0.44 Pa, our simulation results shown in Figs 9 and 10 are the closest to the experimental results reported in [16].

13. In section 3.3: a) The authors assume the platelets will not accumulate on the clot or the wall after the initial platelet aggregation modeled by FCM. What is the justification of this assumption?

Answer: As we responded in question 9(a), we initially placed platelet particles inside the false lumen of the aneurysm such that once the simulation starts, there are sufficient particles to form aggregates inside the false lumen. The platelet particles that do not aggregate will flow downstream. We run the simulation until no new free platelet particles adhere to the existing clot and then we converted the final configuration of the FCM simulation to a phase-field variable as the initial configuration for the phase-field simulation. This method has been successfully implemented in our previous work to predict the development of thrombus in

aortic dissecting aneurysm in a mouse model [6].

b) In Figure 12 (b), since in phase-field results $\phi = 1$ (red) denotes fluid phase, why do we see red regions for the thrombus location in the bottom figure as oppose to the blue ($\phi = 0$) regions shown in the top figure?

Answer: We have updated Figs 11 and 12 such that the colors designation for the FCM simulation results are consistent with those of phase-field simulations.

c) In Figure 13, what is the definition of T? Can the authors also list the physical time unit instead of only the nondimensional time?

Answer: T is the simulation time. We have converted the dimensionless time to the physical time in physical units shown in Fig 13 in the revised manuscript.

d) Can the authors discuss or comment on the effects of the pulsatility on the formation of the thrombus and the deformation of the formed thrombus?

Answer: Please see response to comment #5

Discussion section 4. In general, the authors should provide some analysis of the Results and put them in the context of thrombosis. For example:

a) How does the phase-field model predict thrombosis that is different from previous methods? Can these results be verified in an experiment?

Answer: Most of the previous thrombus models using particle-based methods did not consider the viscoelasticity and permeability, two important properties that determine the deformation and embolism of thrombus under blood flow. The phase-field model, on the other hand, can include both the spatial permeability and the material properties of a thrombus, thereby allowing us to study the effects of thrombus microstructure and material properties on its deformation under different hemodynamic conditions. We emphasize this in the Methods section and Discussion section in the revised manuscript.

The parameters implemented in our model are calibrated by using available experimental data. However, the prediction of deformation and embolism is difficult to validate due to the limitation of the experimental technique.

b) Recent reports indicate that high shear thrombi are much more permeable than coagulation fibrin clots (Kobayashi ISTH 18). Does the inclusion of permeability in your model cause a large difference in

forces on the clot or propensity to evolve differently?

Answer: We thank the reviewer for mentioning this new work. Our sensitivity analysis in Fig 1 in the supporting materials shows that varied permeability causes a large difference in the flow field around the clot.

c) Does the inclusion of a deformable thrombus create embolization where none existed before?

Answer: A deformable thrombus is more biologically realistic and we can estimate the stress exerted on the thrombus by the blood flow and predict the risk of the embolization.

d) Figures 12 and 13 suggest large heterogeneity in thrombus formation in the first 15 seconds, yet Figure 11 indicates that the aneurysm will merely fill in with fibrin after a minute. Since the calculation of Fig 12 and 13 comes at great cost, should we abandon this detail?

Answer: Figs 11(a) and (b) are schematics that show the concept of the FCM simulation and they are not simulation results. Fig 12 (a-c) show the results for the FCM simulation and (d) conversion of the FCM results to the initial configuration for the phase-field simulation. Fig 13 shows the phase-field simulation following the FCM simulation.

ANSWERS FOR REVIEWER #2

We would like to thank **Reviewer #2** for the insightful comments that helped us to improve our manuscript. Please see our answers to your questions below.

Major comments:

1. It is unclear to the reviewer how the platelets (modeled by FCM) are adhering and aggregating, and how their "growth" is considered dynamically in time and in the fluid. In section 2.1 (platelet transport and aggregation) there is no description of how platelets aggregate or adhere to the wall. At what point do they begin to "grow"? How is the spatial distribution of the new larger platelets determined from the distribution of the smaller platelets, especially near the wall? Are the larger platelets stationary? Finally, during the growth stage, how do the authors update the fluid equations surrounding the platelets? Due to lack of details provided on the thrombus formation process makes difficult to fully assess and properly review the study.

Answer: The interactions between the platelets as well as between the platelets and the deposition sites are described by a Morse potential (attractive interactions between platelets) and an exponential repulsion potential (exclusive effects of the platelet particles). The interaction forces between platelets are shear-rate dependent and the force values were validated by using data from four independent experimental studies, including two *in vivo* [5, 6] and two *in vitro* [7, 8] experiments, which measured platelet aggregation at different shear rates. When the activated particles are close to the deposition sites, they are attracted to these sites and adhere to the wall. The adhered platelet particles are considered to be 'stationary' or to 'grow' as part of the clot when their moving distance within in one cardiac cycle is less than 1/100 of their diameter. We have added a new paragraph in the Methods section to describe how the platelets aggregate.

Initially, small particles are uniformly distributed in the aneurysm whereas the distribution of the larger particles depends on the hemodynamics and it is not determined by the distribution of the small particles.

Here, we implement the force coupling method (FCM) where the translational velocity of each platelet (passive or activated) is estimated by the local average of the fluid velocity weighted by a Gaussian kernel function. On the other hand, the fluid surrounding the platelets will be updated by taking into account the platelet interactions by adding an external force term to the governing equation. More detailed information

on the FCM model was added in the last section in Supporting information and it can also be found in [6, 10].

2. The usefulness of going from FCM to a PF model is the heterogeneity of the clot – then the effects of heterogeneity on the mechanics of embolization can be studied; the structure of the thrombus that initially forms is clearly extremely important. Without describing how the platelets are aggregating and which ones have actually aggregated, it is not clear that the phase field model is a correct representation of the clot itself, but rather just a mass of platelets that are flowing through the fluid, in a non-aggregated state. It is possible that this is distinguished somehow but it is not made clear to the reviewer.

Answer: In the revised manuscript, we clarify how the platelets aggregate and grow on the clot in the first paragraph of Methods section.

3. It is unclear to the reviewer where fibrinogen fits into this model. In the methods section, the concentration of fibrinogen is mentioned, and then again in the rheological study section it is used, but there is no evolution equation for fibrinogen for it to exist or interact in the thrombus or at the wall for adhesion.

Answer: Fibrinogen concentration is used only to estimate the volume fraction of the fibrin through $\phi_f = c_{Fbg}/[\rho_{Fbg}\,(0.015\,\log(c_{Fbg}) + 0.13)]$, and we assume the thrombin generated from the activated platelets is enough to convert fibrinogen to fibrin. Thus, we are not modeling the cascade as it makes the model much more complex. The equation for calculating the volume fraction of fibrin was derived experimentally under static conditions [17].

4. The function g1(ϕ) represents a double well potential when phi is in [-1,1] with minima at +/-1; what is the rationale for choosing phi in [0,1] in this model?

Answer: The phase-field variable ϕ is an order parameter and can have any values. In our study, ϕ is correlated to the volume fraction of the blood clot and it is defined in the normalized interval [0 1]. The double well potential can be adjusted based on the defined region of ϕ.

5. The calibration of the permeability of the thrombi were based on experiments with fibrin clots and the empirical formula of Davies is based on fibrous material. The referenced paper (Wufsus et al.) included studies on platelet rich clots where the permeabilities were fit well with a different empirical formula (Ethier). This suggests that the permeabilities of the thrombi modeled in the current study could be significantly underestimated. The authors should instead fit to the platelet-rich clot data or justify their choice for using

the fibrin clots instead of platelet-rich ones.

Answer: There are two clot properties that have to be calibrated in our model: permeability and viscoelasticity. The reviewer is correct that fitting the permeability to platelet-rich clot is more realistic than just fitting to fibrin-rich clot, but we have not been able to find any experiments that measure the viscoelasticity of platelet-rich clot, other than reference [55] which looked at fibrin gel only. Thus, we decided to calibrate both the permeability and viscoelasticity of model against fibrin gel for consistency. Clearly, the same methodology is also valid for platelet-rich clot when experimental data is available. We have mentioned this limitation in the Discussion section.

6. Similarly, the calibrations for the shear modulus are based on rheology experiments performed with fibrin gels while the clots in this study are assumed to be primarily platelets. The section describing how the volume fraction is calculated based on fibrinogen concentration is very confusing. It is not clear what the parameters are in equation 5 or how they relate to the 'VF'. The final sentence in that section relates the relaxation time to a fibrinogen concentration but there is no fibrinogen in the model. This section should be rewritten more carefully and explicitly state what the outcome is.

Answer: Similar to the last comment, we have calibrated the viscoelasticity of thrombi based on fibrin gels because the measurements for the viscoelasticity of platelet-rich clot are not available.

Since the FCM volume fraction cannot determine the composition of a clot in terms of its dominant constituents, here, we consider two important species that contribute to a clot, namely fibrinogen and platelets. Knowing the local concentration of fibrinogen and platelets and using the computed ϕ_{fcm} field, we can estimate the thrombus volume fraction by using $VF = [\ \phi_f(c_{Fbg}) + \phi_p(c_{plat})]\ \phi_{fcm}$, where ϕ_f is the fibrin volume fraction whereas ϕ_p is the volume fraction contribution of platelets [17, 6]. ϕ_f can be computed by an empirical relation $\phi_f =$ $c_{Fbg}/[\rho_{Fbg}\,(0.015\,\log(c_{Fbg}) + 0.13)]$, where c_{Fbg} (mg/mL) is the concentration of fibrinogen and $\rho_{Fbg} =$ $1.4\ g/mL$ is the density of a single fibrinogen molecule [17]. ϕ_p can be evaluated directly based on each platelet's volume and the local platelet number density.

Here we use a normal concentration of fibrinogen in blood when we convert FCM results to VF. We can also add an advection-diffusion equation for F_{bg} and estimate its local concentration.

We have rewritten the paragraph in Methods section to clarify the definition of the VF and how it is calculated.

7. What is the bases for having the AA completely filled with platelets as the initial placement? It seems that one of the benefits of modeling the initial thrombus formation using FCM is that it will lead to more interesting and physiologically relevant thrombus formations. To keep inline with the journal criteria for significant biological insight, it would enhance this study to show a simulation with clots that have grown in the AA that initially was empty.

Answer: The volume of the idealized aneurysm, as shown in Fig 12, is $\sim 7.3\ cm^3$, which is expected to contain 1.095-3.285$\times 10^9$ platelets based on the physiological platelet number density of 150,000-450,000 per microliter of blood. Due to this large number of platelet particles involved, it is computationally prohibitive for us to model all the platelet particles at their physiological size flowing into an aneurysmal expansion and forming clots. Therefore, we simulate a *final-value* problem in lieu of the original *initial-value* problem with significantly fewer particles initially placed in the system which may grow in size upon activation. This approach allows us to use fewer platelets than the physiologic concentration and to grow the size and shape of the clot.

In other words, in vitro experiments are often designed with a test section through which the test fluid flows. Then at the appropriate time, particle are introduced into the system to study pathlines, etc. This is not the case (patho) physiologically. Both the pre-aneurysmal and the enlarging aneurysm always contain blood, and thus always contain large number of platelets (as well as eruthrocytes and leukocytes), hence our formulation is actually not only computationally more tractable, it is physiologically more relevant.

8. The claim is that the timescale of the phase-field modeling covers hours of time (Figure 1), but this claim is not justified by the simulations provided in this paper. Can the authors comment on this?

Answer: Fig 1(a) is just a schematic that illustrates the idea of this framework that can cover multiscale temporal scales and the simulation results could be used to represent or predict a physically long process.

Minor comments:

1. The sensitivity of the surface tension parameter was tested for fixed values of h (and other parameters). The sensitivity was said to be negligible, but it was not clear what tests were used to come to that conclusion.

Is the parameter sensitive as h changes? I don't believe h was ever specified either.

Answer: h is a characteristic length scale of the interface thickness and it is specified in the first paragraph on page 6. We have performed sensitivity analysis of h, as shown in Fig 4 in the supporting materials, and we did not observe notable differences on the deformation of the clots compared to the benchmark value of 1×10^{-3}.

2. In the clot deformation in 2D/3D section, how thick was the z-direction and were the results sensitive to that? As for the two densities and viscosity's, are those for inside and outside the thrombus and which is where?

Answer: We noted that for the 3D simulation, instead of simulating a half spherical geometry, we test a half circular slab with a thickness equal to the width of simulation box in the z-direction, which is periodic. We found that the simulation results are not affected by the thickness in the z-direction.

As shown in Figs 2 and 3, the two different densities and viscosities correspond to densities and viscosities of the blood phase and thrombus phase, respectively. Inside the thrombus, there are two regions, namely the shell and core, which are distinguished only by the permeability.

3. "calibration" would be a better description than "validation" for the permeability model, as it is truly calibration of a model representation.

Answer: Following the reviewer's suggestion, we have updated the terminology.

4. Units. In most of the results sections, the units were left off of the parameters. It would be helpful to at least see units on the permeability.

Answer: Units for the permeability, elastical modulus, and relaxation time were added.

5. In the Modeling thrombin formation with FCM section, the term deposition sites is used, but in the figure, initiation sites is used. Also the reference to figure 11b on the last line of the first paragraph should reference 11c.

Answer: The term "initiation sites" is changed to "deposition sites" in Fig 11(a) and reference to Fig 11(b) is changed to Fig 11(c) in the revised manuscript.

6. What is the upstream distribution of platelets during the dynamic simulation?

Answer: It is known that margination of platelets occurs due to collisions with red blood cells in the

blood vessels, which has been studied extensively experimentally and theoretically, for straight channels and idealized vessels. In this work, as we did not simulate the red blood cells explicitly in the blood flow, when we insert particles at the inlet, the distribution of particles follows a master profile for platelet distribution to account for their margination in the aorta, as introduced in [18]. We have added this explanation into the main text of Methods section.

References

[1] Tong J, Holzapfel GA. Structure, mechanics, and histology of intraluminal thrombi in abdominal aortic aneurysms. Annals of Biomedical Engineering. 2015;43(7):1488–1501.

[2] Schriefl A, Collins M, Pierce D, Holzapfel GA, Niklason L, Humphrey J. Remodeling of intramural thrombus and collagen in an Ang-II infusion ApoE-/- model of dissecting aortic aneurysms. Thrombosis Research. 2012;130(3):e139–e146.

[3] Mountrakis L, Lorenz E, Hoekstra A. Where do the platelets go? A simulation study of fully resolved blood flow through aneurysmal vessels. Interface Focus. 2013;3(2):20120089.

[4] Di Achille P, Tellides G, Humphrey J. Hemodynamics-driven deposition of intraluminal thrombus in abdominal aortic aneurysms. International Journal for Numerical Methods in Biomedical Engineering. 2017;33(5):e2828.

[5] Begent N, Born G. Growth rate in vivo of platelet thrombi, produced by iontophoresis of ADP, as a function of mean blood flow velocity. Nature. 1970;227:926–930.

[6] Yazdani A, Li H, Bersi MR, Di Achille P, Insley J, Humphrey JD, et al. Data-driven modeling of hemodynamics and its role on thrombus size and shape in aortic dissections. Scientific reports. 2018;8(1):2515.

[7] Westein E, van der Meer AD, Kuijpers MJ, Frimat JP, van den Berg A, Heemskerk JW. Atherosclerotic geometries exacerbate pathological thrombus formation poststenosis in a von Willebrand factor-dependent manner. Proceedings of the National Academy of Sciences. 2013;110(4):1357–1362.

[8] Li M, Ku DN, Forest CR. Microfluidic system for simultaneous optical measurement of platelet aggregation at multiple shear rates in whole blood. Lab on a Chip. 2012;12(7):1355–1362.

[9] Pivkin IV, Richardson PD, Karniadakis G. Blood flow velocity effects and role of activation delay time on growth and form of platelet thrombi. Proceedings of the National Academy of Sciences. 2006;103(46):17164–17169.

[10] Yazdani A, Li H, Humphrey JD, Karniadakis GE. A general shear-dependent model for thrombus formation. PLoS computational biology. 2017;13(1):e1005291.

[11] Yang Z, Dong S. Multiphase flows of N immiscible incompressible fluids: An outflow/open boundary condition and algorithm. Journal of Computational Physics. 2018;366:33–70.

[12] Dong S. Multiphase flows of N immiscible incompressible fluids: A reduction-consistent and thermodynamically-consistent formulation and associated algorithm. Journal of Computational Physics. 2018;361:1–49.

[13] Tierra G, Pavissich JP, Nerenberg R, Xu Z, Alber MS. Multicomponent model of deformation and detachment of a biofilm under fluid flow. Journal of The Royal Society Interface. 2015;12(106):20150045.

[14] Xu S, Alber M, Xu Z. Three-phase Model of Visco-elastic Incompressible Fluid Flow and its Computational Implementation. Communications in Computational Physics Available at: https://www researchgate net/publication/322959186_Three-phase_Model_of_Visco-elastic_ Incompressible_Fluid_Flow_and_its_Computational_Implementation. 2018;.

[15] Xu S, Xu Z, Kim OV, Litvinov RI, Weisel JW, Alber M. Model predictions of deformation, embolization and permeability of partially obstructive blood clots under variable shear flow. Journal of the Royal Society Interface. 2017;14(136):20170441.

[16] van Kempen TH, Bogaerds AC, Peters GW, van de Vosse FN. A constitutive model for a maturing fibrin network. Biophysical journal. 2014;107(2):504–513.

[17] Wufsus A, Macera N, Neeves K. The hydraulic permeability of blood clots as a function of fibrin and platelet density. Biophysical Journal. 2013;104(8):1812–1823.

[18] Aarts P, Van Den Broek S, Prins GW, Kuiken G, Sixma JJ, Heethaar RM. Blood platelets are con-

centrated near the wall and red blood cells, in the center in flowing blood. Arteriosclerosis: An Official Journal of the American Heart Association, Inc. 1988;8(6):819–824.
